# Navigating Complex Cardiac Defects: A Neonatal Case of Pentalogy of Fallot With Vascular Anomalies Identified via Cardiac CT Angiography

**DOI:** 10.7759/cureus.80338

**Published:** 2025-03-10

**Authors:** Luis Alberto Solís García, Oscar Antonio Regalado Morales, Marcelo Valdés Hernández, Samuel Iván Espinoza Tristán, Martha Catalina Carrillo Tamez

**Affiliations:** 1 Radiology, Hospital Regional Institute of Security and Social Services for State Workers (ISSSTE) Monterrey, Monterrey, MEX

**Keywords:** cardiac computed tomography angiography, cardiovascular radiology, pediatric cardiologist, pediatric radiology, pentalogy of fallot

## Abstract

The pentalogy of Fallot stands out as one of the rarest and most complex congenital heart defects. Pulmonary artery stenosis, a ventricular septal defect, an overriding aorta, right ventricular hypertrophy, and atrial septal communication characterize it. Due to its complexity and associated malformations, advanced imaging methods, such as cardiac CT angiography, have become essential for proper characterization.

In this study, we report the case of a 35-week gestation newborn diagnosed with pentalogy of Fallot and additional vascular malformations, identified through cardiac CT angiography.

## Introduction

Congenital heart defects are a significant global health concern, with an incidence ranging from 8 to 12 cases per 1,000 live births [1]. Among major congenital malformations, these account for up to 85% of cases, making them a leading cause of morbidity and mortality [2]. For diagnostic purposes, they are classified into cyanotic and non-cyanotic heart defects. The tetralogy of Fallot is the most common cyanotic congenital heart defect, accounting for 7% to 10% of all congenital heart conditions [3]. While the components of this defect are well-defined, associated malformations are frequently observed, adding to its complexity. This highlights the importance of detailed descriptions across different imaging modalities and leads to using other terms, such as the pentalogy of Fallot [4].

In this study, we report the case of a 35-week-old newborn who was diagnosed with pentalogy of Fallot and additional vascular malformations through cardiac CT angiography.

This article was previously presented as a poster at the Annual meeting (58th) of the European Society of Pediatric Radology.

## Case presentation

The patient is a premature male newborn delivered at 35 weeks of gestation, born to a mother with a history of type 2 diabetes treated with metformin during pregnancy. The neonate weighed 2,610 grams at birth and measured 48 cm in length. On physical examination, signs of respiratory distress were observed, including intercostal retractions, grunting, thoracoabdominal dissociation, and xiphoid retraction. Additionally, a grade III/IV heart murmur was detected, along with diffuse cyanosis, predominantly in the extremities and perioral region during crying. Vital signs at birth included a heart rate of 150 bpm, respiratory rate of 50 breaths per minute, oxygen saturation of 85%, and blood pressure of 64/27 mmHg. Laboratory tests were also performed, which showed elevated hematocrit secondary to chronic hypoxia (Table 1).

**Table 1:** Initial laboratory tests.

Laboratory Test	Result	Unit	Reference
Leukocytes	9.2	10^3/uL	4.5-10.5
Hemoglobin	18.8	g/dL	14-18
Hematocrit	55.6	%	42-52
Platelets	171	10^3/uL	140-400
Glucose	94	mg/dL	74-106
Creatinine	0.7	mg/dL	0.50-1.30

Imaging findings

**Figure 1:**
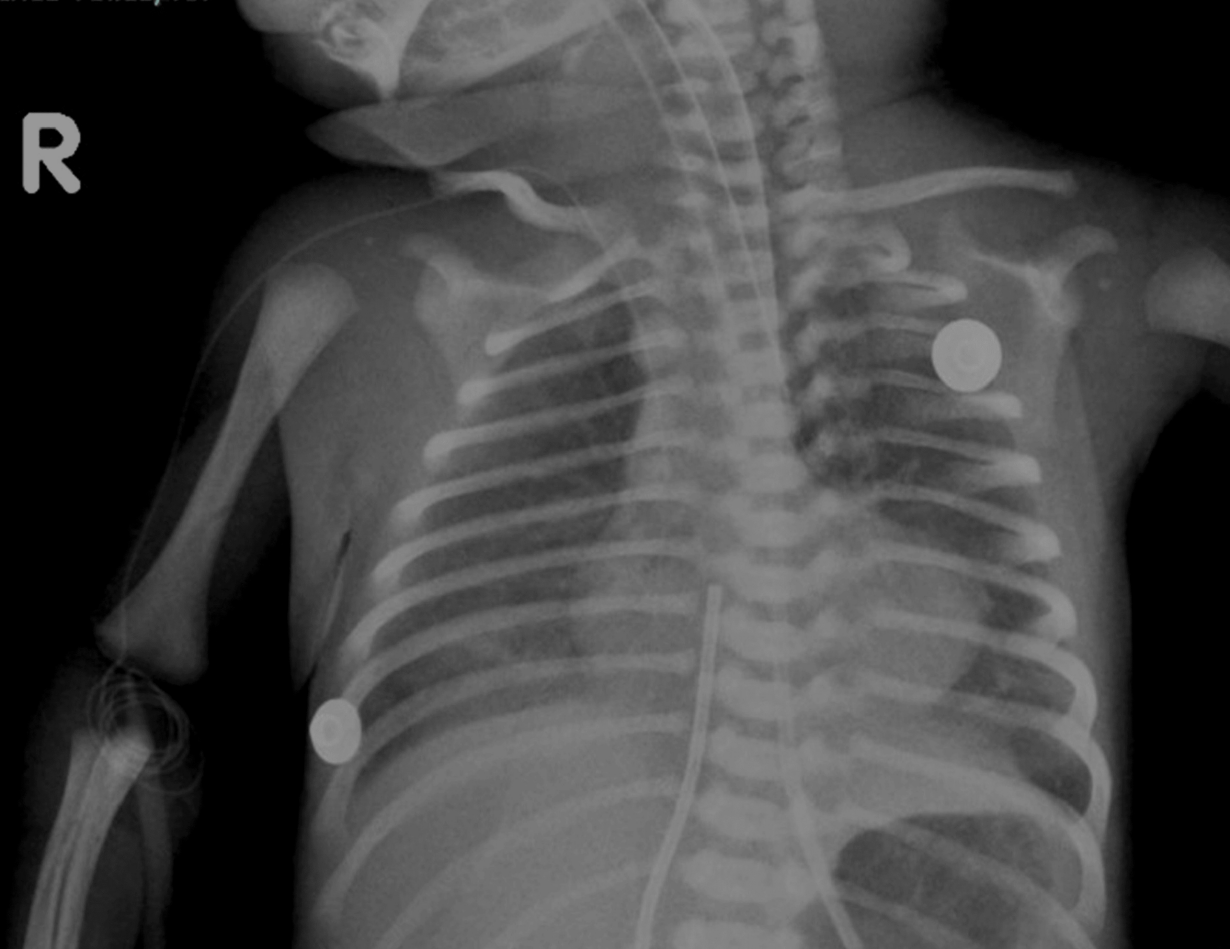
Chest radiograph. The initial radiograph shows elevation of the cardiac apex, a boot-shaped heart sign, and interstitial infiltrates in both lungs.

A chest X-ray was performed, revealing cardiomegaly predominantly affecting the right-sided heart chambers, with elevation of the cardiac apex, giving the characteristic "boot-shaped" appearance. Additionally, bilateral reticular infiltrates were observed in the pulmonary parenchyma (Figure 1). In the following days, a pediatric cardiology consultation was requested, during which an echocardiogram was performed. The findings included atrial septal defect (ASD), ventricular septal defect (VSD), overriding aorta, and patent ductus arteriosus (PDA).

**Figure 2:**
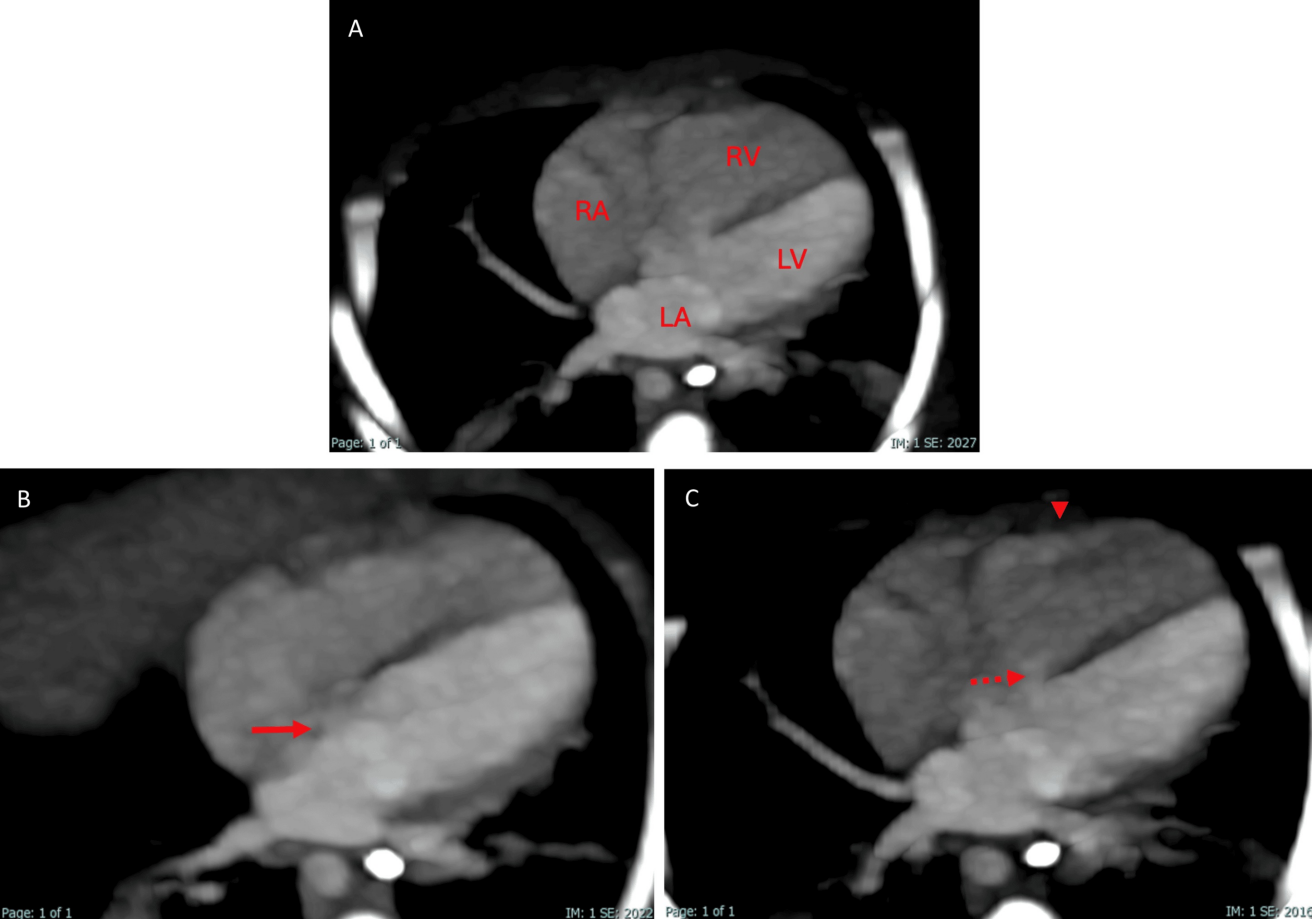
Cardiac CT angiography in oblique axial cuts at the level of the heart. (A) The image shows the right auricle (RA), left auricle (LA), right ventricle (RV), and left ventricle (LV). (B) At other levels of the plane, an atrial septal defect (arrow) is demonstrated. (C) Additionally, the ventricular septal defect (dashed arrow) and right ventricular hypertrophy (arrowhead) are observed.

**Figure 3:**
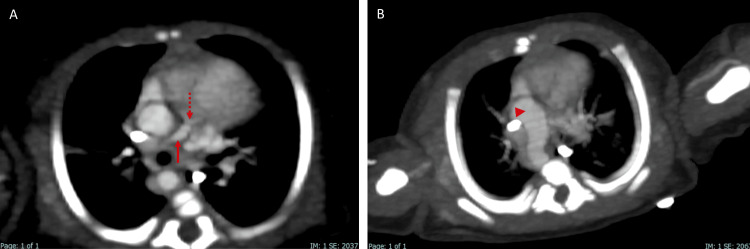
Cardiac CT angiography in oblique axial at the level of the heart. (A) The image shows the right ventricular outflow tract with hypoplasia of the pulmonary artery (arrow) and pulmonary atresia (dashed arrow). (B) Additionally, the right-sided aortic arch (arrowhead) is observed.

**Figure 4:**
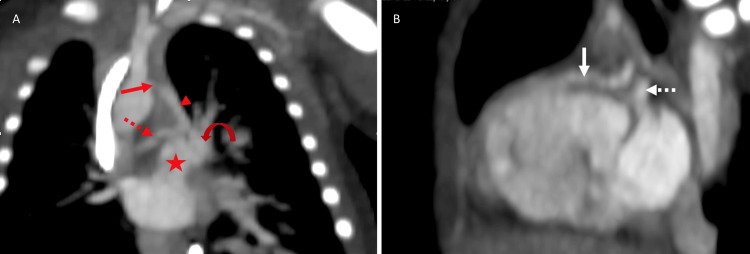
Cardiac CT angiography in coronal (A) and sagittal (B) plane. (A) The coronal image shows hypoplasia of the pulmonary trunk (star) and its left (curved arrow) and right (red dashed arrow) branches. Additionally, there is persistence of the ductus arteriosus (red arrowhead) with narrowing towards the aortic arch (red arrow), which reaches the right pulmonary artery branch. (B) The sagittal image depicts the outflow tract of the left ventricle (white arrow) and the presence of an aortopulmonary collateral (white dashed arrow).

**Figure 5:**
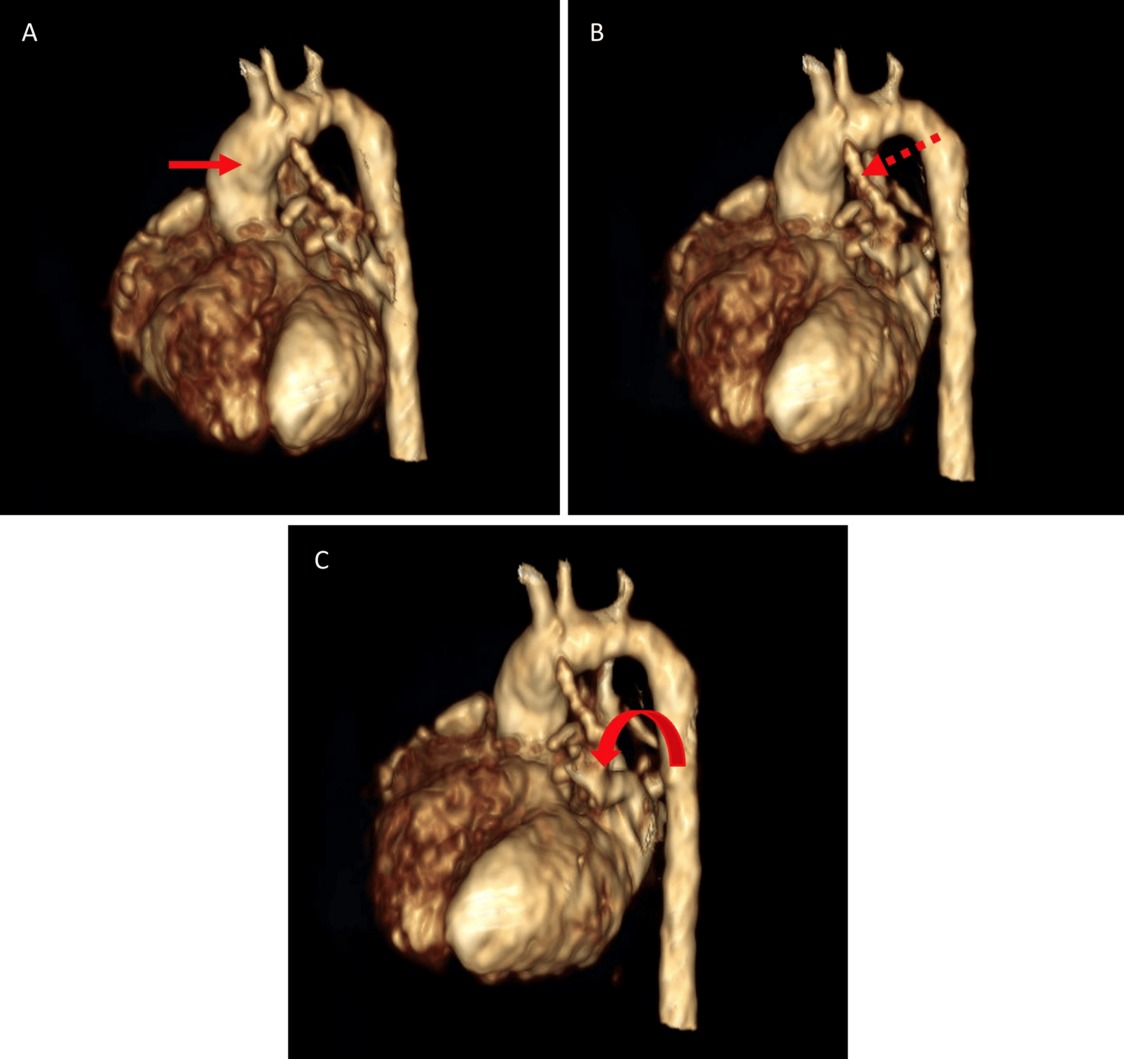
3D reconstruction of cardiac CT angiography. This image shows the ascending aorta (red arrow in A), persistent ductus arteriosus (dashed arrow in B), and hypoplasia of the pulmonary trunk and arteries (curved arrow in C).

Based on these findings, a cardiac CT angiography was performed in the weeks following the patient's stabilization to assess the severity of the disease. It revealed situs solitus of the atria, bronchi, and abdomen, with concordant atrioventricular and ventriculoarterial connections. The four-chamber view showed a VSD and an ostium secundum-type ASD, as well as right ventricular hypertrophy (Figure 2). At the level of the great vessels, pulmonary valve atresia was identified, along with hypoplastic pulmonary arteries, aortopulmonary collateral arteries, a right-sided aortic arch, and a PDA (Figures 3-5). These findings were consistent with the pentalogy of Fallot with pulmonary valve atresia.

The patient was subsequently evaluated by pediatric surgery, where it was determined that surgical intervention was not feasible due to poor prognostic.

## Discussion

Tetralogy of Fallot occurs in 4-5 per 10,000 live births, making it the most common cyanotic congenital heart defect. It consists of pulmonary artery stenosis, a VSD, an overriding aorta, and right ventricular hypertrophy [3]. Although these components are well-defined, associated malformations such as a right-sided aortic arch, an aberrant origin of coronary arteries, or an ASD are often observed [5]. When the latter is present, it results in a variant known as the pentalogy of Fallot, an extremely rare condition with an estimated incidence of 0.006% in the general population, in comparison with tetralogy of Fallot, which has an incidence of 0.34% [6].

The possible origin of the tetralogy of Fallot and its variants is attributed to a defective partitioning of the conotruncus and incomplete rotation, resulting in an unequal size of the aorta and pulmonary artery. This defect causes a displacement of the conal septum, which shifts anteriorly and superiorly along with the infundibular septum. This movement is responsible for the four main anatomical characteristics defining the tetralogy of Fallot [3].

Tetralogy of Fallot and its variants affect males and females similarly and occurs in isolation in most cases (75-80%). However, in 20-25% of cases, it is associated with genetic syndromes, the most common being Down syndrome and DiGeorge syndrome [7].

Symptoms may appear at birth or remain virtually asymptomatic in some cases. Most patients present with cyanosis during the neonatal period; however, sometimes, the only clinical finding may be a heart murmur. During childhood, hypercyanotic episodes can occur, often triggered by agitation or irritability and accompanied by tachypnea. In severe cases, these episodes can lead to syncope, requiring immediate intervention to stabilize the patient [8].

The described symptoms originate from the combination of a VSD and pulmonary artery stenosis. The restriction of blood flow to the pulmonary circulation increases right ventricular pressure, leading to a right-to-left shunt through the septal defect. As a result, deoxygenated blood enters the systemic circulation, causing cyanosis. This pressure difference becomes more pronounced during episodes of agitation or crying, further worsening the symptoms. Additionally, the heart murmur is secondary to pulmonary stenosis and varies in intensity depending on its severity, becoming absent in cases of severe stenosis [3].

Imaging studies are fundamental in the evaluation of these patients. A chest X-ray is usually the initial study, where cardiomegaly and elevation of the cardiac apex secondary to right ventricular hypertrophy can be observed, giving the characteristic "boot-shaped" appearance [5].

Echocardiography is the first-line study and is essential for diagnosis, from the prenatal stage to identifying complications throughout the patient’s life. However, it has certain limitations, such as detailed evaluation of the aorta, pulmonary arteries, pulmonary veins, and coronary arteries [9].

To overcome these limitations, other imaging modalities, such as computed tomography (CT), have gained relevance. CT provides superior spatial resolution and greater anatomical detail of vascular and non-vascular structures, making it especially useful in complex cardiac defects like tetralogy of Fallot, given its significant variability. This technique accurately evaluates the location of pulmonary stenosis and detects associated anomalies such as ASDs, right-sided aortic arch, left superior vena cava, and coronary artery anomalies. Additionally, it is valuable for identifying airway alterations, lung issues, and other major vessel abnormalities, providing key information for surgical planning. However, it is important to acknowledge that CT has certain limitations, including radiation exposure, the need for sedation in neonates, and the fact that, unlike echocardiography, it cannot be performed at the patient’s bedside [10].

## Conclusions

The pentalogy of Fallot is an extremely rare and complex congenital malformation with significant implications for the health and development of newborns. This article highlights the usefulness of advanced imaging studies, such as cardiac CT angiography, in the detailed characterization of the components of the pentalogy of Fallot and associated malformations, such as pulmonary artery hypoplasia, right aortic arch, and aortopulmonary collateral vessels. These findings emphasize the value of CT as a more comprehensive diagnostic tool compared to traditional imaging studies, such as echocardiography, as it allows for the evaluation not only of the heart but also of vascular and non-vascular structures, which play a key role in clinical management planning. In this case, the multiple malformations detected in the CT study made it impossible to offer surgical treatment, highlighting both the severity and variability of this condition.
